# Does month of birth influence colorectal cancer prognosis?

**DOI:** 10.1007/s00423-023-03161-3

**Published:** 2023-10-26

**Authors:** José Martín-Arévalo, David Moro-Valdezate, Vicente Pla-Martí, Stephanie García-Botello, Pablo Moya-Marcos, Ana Izquierdo-Moreno, Leticia Pérez-Santiago, David Casado-Rodrigo, Susana Roselló-Keränen, Alejandro Espí-Macías

**Affiliations:** 1 Colorectal Surgery Unit, Department of General and Digestive Surgery, INCLIVA Biomedical Research Institute, Hospital Clínico Universitario de Valencia, University of Valencia, Av. Blasco Ibáñez, 17, 46010 Valencia, Spain; 2 Department of Surgery, University of Valencia, Valencia, Spain; 3 Department of Medical Oncology, INCLIVA Biomedical Research Institute, Hospital Clínico Universitario de Valencia, Valencia, Spain

**Keywords:** Colorectal cancer, Prognosis, Month of birth, Survival

## Abstract

**Purpose:**

The main aim of this study was to identify a possible association between month of birth of colorectal cancer (CRC) patients and overall survival (OS) or disease-free survival (DFS).

**Methods:**

This observational study included all consecutive adult patients diagnosed with CRC undergoing oncological surgery from January 2005 to December 2019 with a minimum follow-up of 10 years. The outcome variables were locoregional recurrence, death due to cancer progression, OS and DFS. Non-supervised learning techniques (K-means) were conducted to identify groups of months with similar oncologic outcomes. Finally, OS and DFS were analysed using Kaplan–Meier and Cox regression tests. The model was calibrated with resampling techniques and subsequently a cross-validation was performed.

**Results:**

A total of 2520 patients were included. Three birth month groups with different oncologic outcomes were obtained. Survival analysis showed between-group differences in OS (*p* < 0.001) and DFS (*p* = 0.03). The multivariable Cox proportional hazards model identified the clusters obtained as independent prognostic factors for OS (*p* < 0.001) and DFS (*p* = 0.031).

**Conclusion:**

There is an association between month of birth and oncologic outcomes of CRC. Patients born in the months of January, February, June, July, October and December had better OS and DFS than those born in different months of the year.

**Supplementary Information:**

The online version contains supplementary material available at 10.1007/s00423-023-03161-3.

## Introduction

It is widely acknowledged that genetics, lifestyle, and environmental factors play a significant role in the development of chronic diseases and longevity. However, emerging evidence suggests that factors acting during pregnancy or the perinatal period can also have a significant impact on individual risk of developing chronic diseases, and decrease life expectancy in adulthood. These factors include nutritional status, inflammatory response, exposure to infections, and other environmental influences such as temperature or sunlight exposure [1, 2]. Some studies have shown that infants born in the summer have higher birth weights than those born in other seasons, and there is evidence that birth weight is associated with adult health outcomes. This suggests that season of birth can contribute to the development of adult diseases and aging-related processes [3, 4].

Research has also shown that the month of birth can influence the risk of cancer development. Several studies in the United Kingdom have found month-of-birth patterns for acute leukaemia, lymphoma, glioma and osteosarcoma, with birth clusters in autumn and winter months [5, 6]. Other studies have also observed seasonality of birth for other types of tumours, such as Hodgkin´s lymphoma in the Danish population under 20 years of age, brain tumours in adults in USA, skin cancer in Italy, lung cancer in China and breast cancer in Norway [7–10].

To date, a study by Francis et al. is the only one to suggest an association between month of birth and risk of developing colorectal cancer (CRC) [11]. However, the relationship between month of birth and CRC prognosis has not yet been studied. CRC prognosis is influenced by factors such as neoplasm stage and grade, patient age and overall health, and tumour response to therapy, in addition to factors such as nutrition, chronic diseases and psychological stress.

The main objective of this study was to identify a possible association between the month of birth of patients diagnosed with CRC and overall survival (OS) or disease-free survival (DFS).

## Material and methods

### Study design and patients

This retrospective observational study comprised a cohort of all consecutive adult patients diagnosed with CRC who underwent oncological surgery from January 2005 to December 2019. Surgery was performed by the Colorectal Surgery Unit of a tertiary university institution (University Clinic Hospital of Valencia, Spain). This unit has advanced accreditation from the Spanish Society of Coloproctology. The tumours were staged according to the 8^th^ edition of the American Joint Committee on Cancer classification. The study was conducted in accordance with STROBE recommendations. Inclusion criteria were age over 18 years, birthplace in Valencia region, histologic diagnosis of colon or rectal cancer, oncologic surgical resection, tumour stages I–IV and a minimum follow-up of 36 months. Exclusion criteria were appendicular tumour, anal tumour, and local rectal resection. There is evidence suggesting a relationship between seasonal variation and postoperative surgical outcomes, mainly influenced by meteorological conditions and the characteristics of each health care system (staffing, hospital resources or pressure on health care system) [12, 13]. Therefore, the patients who died during the immediate postoperative period (30 days after surgery) were excluded to avoid possible biases. The patients who died from causes other than cancer progression were also excluded.

### Study endpoints and outcome variables

The aim of this study was to identify a possible association between month of birth and tumour recurrence or death due to cancer progression in CRC patients. The qualitative outcome variables were recurrence and death due to disease progression. Recurrence was defined as any tumour reappearance in the same location or in distant organs, and it was diagnosed by clinical signs, increased serum carcinoembryonic antigen (CEA) levels and complementary examinations (colonoscopy, chest/abdominal computed tomography (CT) scan, magnetic resonance imaging or positron emission tomography scan). If imaging studies were conclusive, biopsy was not considered necessary. The quantitative outcome variables were OS and DFS. OS was defined as the time in months from surgery to the death of the patient due to oncologic progression, while DFS was defined as the time in months from surgery to confirmed cancer recurrence.

### Data source and study variables

Data were collected in an institutional standardised relational database. Complete medical history and hospital medical records were reviewed and subsequently analysed. The study variables were age, sex, month of birth, American Society of Anaesthesiologists (ASA) score, tumour location (ascendant colon, transverse colon, splenic flexure of the colon, descendent colon, sigmoid colon or rectum), histological differentiation grade, peritumoral invasion, TNM classification, and tumour stage according to the 8^th^ edition of the *American Joint Committee on Cancer* (2018). Patient follow-up was performed by the Department of Medical Oncology and the Colorectal Surgery Unit in the outpatient clinic at 1 month and 3 months, then every 6 months during the first 2 years, and thereafter annually for a minimum of 3 years. The periodic controls included serum CEA levels, rigid proctoscopy in rectal cancer and chest/abdominal CT scan performed upon elevated CEA levels or at yearly intervals if the latter was normal. A colonoscopy was made 1 year after resection and afterwards every 3–4 years. The minimum follow-up period for patients without recurrence or death due to cancer progression was 10 years.

### Statistical analysis

A descriptive analysis was made of each variable of the sample. Normality of quantitative variables was determined through Kolmogorov–Smirnov test. Quantitative variables were expressed as median and range, and qualitative variables as percentages. Fisher’s exact test or χ^2^ tests were used to find possible differences between qualitative variables, while the Mann–Whitney U test was used for quantitative variables. To analyse the series, a data table was created relating month of birth with number of cases, recurrences, and deaths due to disease progression. A cluster classification (a type of unsupervised learning technique) was used to identify a possible pooling pattern of similar oncologic outcomes according to month of birth. The month groups were created using the unsupervised automatic classification technique *k-means*. The optimal number of clusters was determined analyzing the Euclidean distance between months. In this clustering the goal was to minimize the distance between the months within each group while maximizing the Euclidean distance between groups. Next, we conducted a descriptive analysis of the clusters of months and possible between-group differences were studied. Finally, OS and DFS were analysed using Kaplan–Meier and Cox regression tests. The Harrell's C-index was computed to evaluate the discriminative capacity of OS and DFS of Cox regression models and the accuracy of predictions concerning time-to-event outcomes, such as survival times. This index quantified the likelihood that, when selecting a random pair of individuals, the one with the higher predicted risk score will experience the event of interest at an earlier time. The calibration and validation of Cox proportional hazards models for OS and DFS were driven. Model calibration involved the use of a resampling (bootstrap) technique with 200 replicates. Subsequently, a cross-validation of the model was performed. Metrics of resampling techniques were calculated for both models.

*P* value < 0.05 was considered statistically significant. Statistical analysis was carried out with SPSS® Statistics Version 25 for Windows and R® Version 4.2.1 for Windows.

## Results

### Descriptive analysis

A total of 2520 patients diagnosed with CRC were included in the study over a period of 15 years. Patients and tumour characteristics are outlined in Table 1. Median patient age was 72 years (range = 69) and 993 patients (39.4%) were female. The tumour was more frequently located in the rectum (33.4%), ascendant colon (27.7%) and sigmoid colon (25.0%). Most patients included in the study presented tumour stage II (30.8%) or stage III (29.0%). During the follow-up period cancer recurrence was found in 19.6% of patients, while the mortality rate due to oncological progression was 22.5%.

**Table 1:** Patient and tumour characteristics of the series

Variable	*n* = 2520
Age (years)	72 (69)
Sex	
Male	1527 (60.6)
Female	993 (39.4)
ASA score	
I	143 (5.7)
II	1129 (44.8)
III	1159 (46.0)
IV	89 (3.5)
Tumour location	
Ascendant colon	697 (27.7)
Transverse colon	118 (4.7)
Splenic flexure of the colon	158 (6.3)
Descendent colon	76 (3.0)
Sigmoid colon	630 (25.0)
Rectum	841 (33.4)
Histologic differentiation grade	
Low	2326 (92.3)
High	188 (7.5)
Unknown	6 (0.2)
Peritumoral invasion	
Lymphovascular	740 (29.4)
Perineural	777 (30.8)
Local invasion (AJCC)	
pTx	64 (2.6)
pTis	64 (2.6)
pT0	25 (1.0)
pT1	206 (8.2)
pT2	442 (17.5)
pT3	1137 (45.1)
pT4	582 (23.1)
Lymph node metastases (AJCC)	
pNx	74 (2.9)
pN0	1493 (59.2)
pN1	622 (24.7)
pN2	331 (13.2)
Distant metastases (AJCC)	
Mx	22 (0.9)
M0	2112(83.8)
M1	386 (15.3)
Tumour stage (AJCC)	
0	91 (3.6)
I	536 (21.3)
II	775 (30.8)
III	732 (29.0)
IV	386 (15.3)
Overall Survival (months)	71 (216)
Disease-Free Survival (months)	61 (217)
Mortality due to cancer progression	548 (22.5)
Recurrence	495 (19.6)

*ASA* American society of anaesthesiologists

*AJCC* American joint committee on cancer, 8^th^ edition (2018)

Statistics presented as median (range) or *n* (%)

### Pooling months of birth according to recurrence and mortality rates

**Fig. 1:**
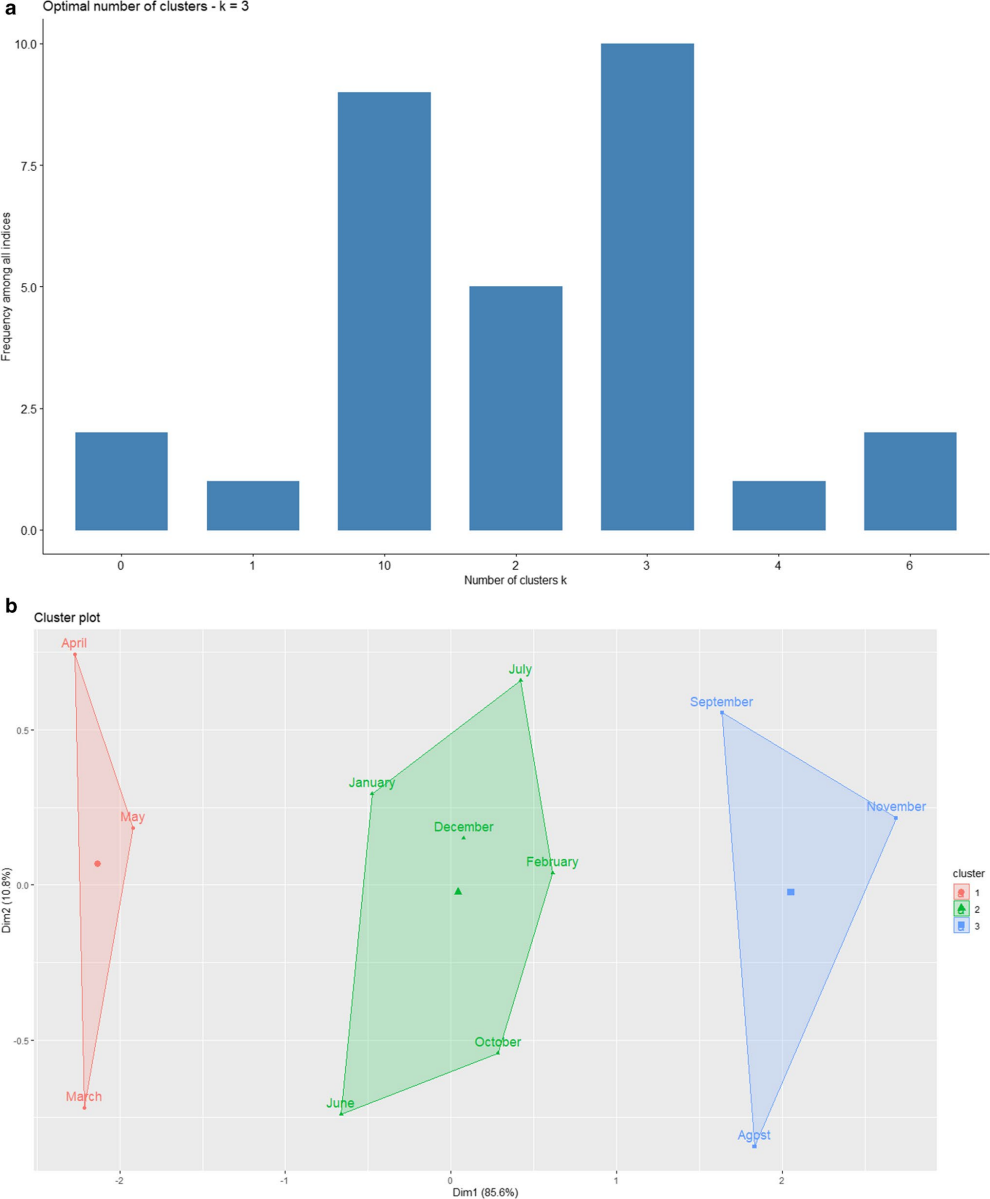
Cluster analysis. **A** Optimal number of cluster analysis. **B** Cluster plot of birth months according to recurrence and mortality outcomes

Three clusters were determined as the optimal number of classification clusters for this data set, as it is depicted in Fig. 1A. Non-supervised learning techniques (K-means) were used to pool months with similar outcome variables, and three different month groups were clearly identified: *Group 1* = March, April and May, *Group 2* = January, February, June, July, October and December and *Group 3* = August, September and November (Fig. 1B).

### Month-of-birth cluster analysis

Patient and tumour characteristics after month-of-birth clustering are outlined in Table 2. No significant differences between the three groups were found in study variables. However, group 2 achieved longer DFS than groups 1 and 3 (*p* = 0.022). Thereafter, group 2 revealed a lower rate of mortality due to cancer progression (*p* < 0.001) and a lower recurrence rate (*p* = 0.044) than the other groups.

**Table 2:** Patient and tumour characteristics after month-of-birth clustering

Variable	Group 1 (*n* = 710)	Group 2 (*n* = 558)	Group 3 (*n* = 1252)	*p*-value
Age (years)	72 (65)	72 (67)	71 (67)	0.559
Sex				
Male	409 (59.7)	325 (60.6)	737 (60.8)	0.891
Female	276 (40.3)	211 (39.4)	475 (39.2)	
ASA score				
I	42 (5.9)	30 (5.4)	71 (5.7)	0.240
II	330 (46.5)	247 (44.3)	552 (44.1)	
III	308 (43.4)	265(47.5)	586 (46.8)	
IV	30 (4.2)	16 (2.9)	43 (3.4)	
Tumour location				
Ascendant colon	186 (26.2)	156 (28)	355 (28.4)	0.678
Transverse colon	35 (4.9)	18 (3.2)	65 (5.2)	
Splenic flexure of the colon	39 (5.5)	35 (6.3)	84 (6.7)	
Descendent colon	29 (4.1)	19 (3.4)	28 (2.2)	
Sigmoid colon	187 (26.3)	133 (23.9)	310 (24.8)	
Rectum	234 (33)	197 (35.3)	410 (32.7)	
Histologic differentiation grade				
Low	653 (92)	514 (92.1)	1159 (92.6)	0.460
High	56 (7.9)	44 (7.9)	88 (7)	
Unknown	1 (0.1)	–	5 (0.4)	
Peritumoral invasion				
Lymphovascular	221 (31.1)	149 (26.7)	370 (29.6)	0.224
Perineural	170 (23.9	130 (23.9)	296 (23.6)	0.964
Local invasion (AJCC)				
pTx	18 (2.6)	9 (1.6)	34 (3)	0.437
pTis	22 (3.1)	14 (2.5)	28 (2.2)	
pT0	8 (1.1)	2 (0.4)	15 (1.2)	
pT1	54 (7.6)	47 (8.4)	105 (8.4)	
pT2	112 (15.8)	107 (19.2)	223(17.8)	
pT3	335 (47.2)	251 (45)	551 (44)	
pT4	161 (22.6)	128 (22.9)	293 (23.4)	
Lymph node metastases (AJCC)				
pNx	37 (5.2)	21 (3.8)	42 (3.4)	0.181
pN0	412 (58)	336 (60.2)	719 (57.4)	
pN1	171 (24.1)	128 (22.9)	323 (25.8)	
pN2	90 (12.7)	73 (13.1)	168 (13.4)	
Distant metastases (AJCC)				
Mx	7 (1)	7 (1.3)	8 (0.6)	0.511
M0	593 (83.6)	544 (85.2)	1044 (83.3)	
M1	110 (15.5)	76 (13.6)	200 (16)	
Tumour stage (AJCC)				
0	26 (3.7)	19 (3.4)	46 (3.7)	0.661
I	139 (19.6)	126 (22.6)	271 (21.6)	
II	228 (32.1)	183 (32.8)	364 (29.1)	
III	207 (29.2)	154 (27.6)	371 (29.6)	
IV	110 (15.5)	76 (13.6)	200 (15)	
Overall Survival (months)	67 (216)	75 (216)	72 (215)	0.054
Disease-Free Survival (months)	56 (217)	66 (217)	62 (216)	**0.022**
Mortality due to cancer progression	178 (25.1)	90 (16.1)	280 (22.4)	** < 0.001**
Recurrence	159 (22.4)	94 (16.8)	242 (19.3)	**0.044**

Bold entries highlighted statistical significancy

Group 1: March, April and May. Group 2: January, February, June, July, October and December. Group 3: August, September and November

*ASA* American society of anaesthesiologists; *AJCC* American joint committee on cancer, 8^th^ edition (2018)

Statistics presented as median ± standard deviation or *n* (%)

**Fig. 2:**
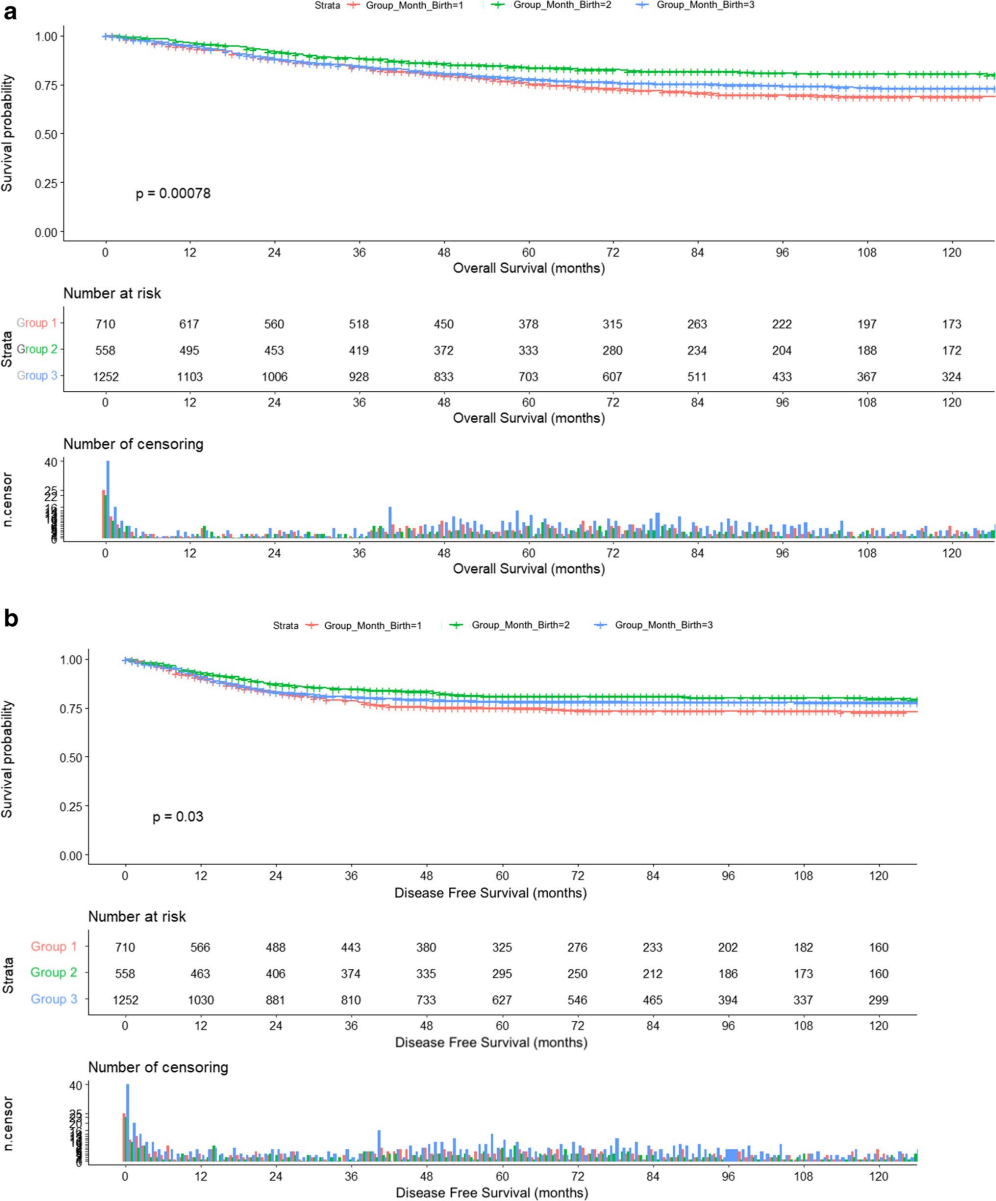
Survival curves of the Kaplan–Meier analysis. **A** Overall survival curves. **B** Disease-free survival curves

**Fig. 3:**
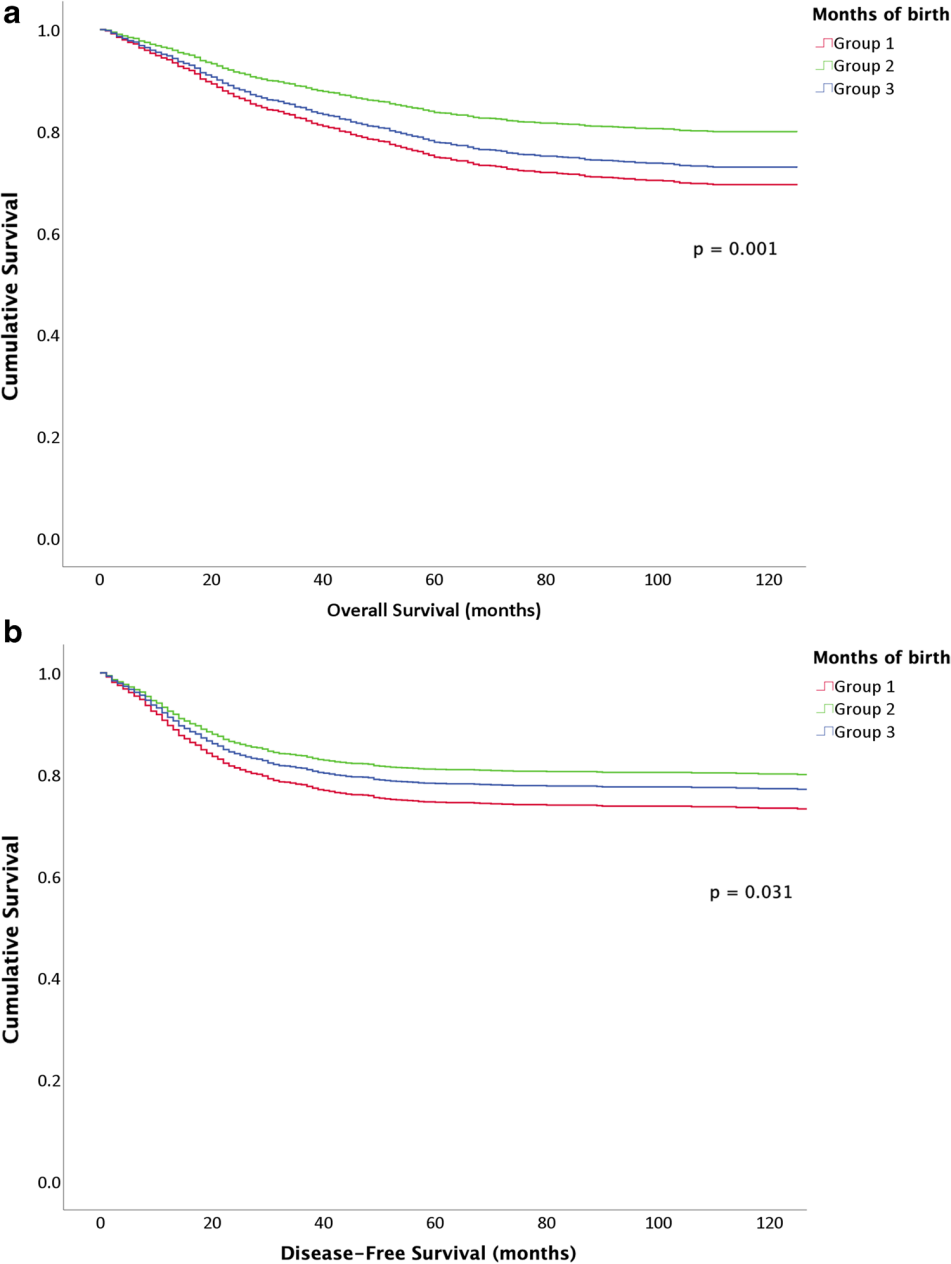
Survival curves of the multivariable Cox proportional hazards model. **a** Overall survival curves. **b** Disease-free survival curves

Survival analysis showed between-group differences in OS (*p* < 0.001) and DFS (*p* = 0.03). In the OS analysis, Group 2 had better OS (180.99 ± 3.43 months) than Group 1 (161.28 ± 3.6 months; *p* = 0.012) or Group 3 (167.54 ± 2.45 months; *p* < 0.001). Similarly, DFS analysis between clusters revealed better DFS for Group 2 (177.69 ± 3.65 months) than Group 1 (164 ± 3.6 months; *p* = 0.047) or Group 3 (171.9 ± 2.52 months; *p* = 0.006) (Fig. 2). The multivariable Cox proportional hazards model only included tumour stage and month-of-birth groups. This model reported that the clusters obtained were independent prognostic factors for OS (*p* < 0.001) and DFS (*p* = 0.031) (Fig. 3). Harrell's C-index for OS model was 0.81 and Somers' Dxy rank was 0.61, while Harrell's C-index for DFS model was 0.7 and Somers' Dxy rank was 0.32. Metrics for both models are available in supplementary files (Calibration graphics and tables with metrics for OS and DFS).

Comparing the mortality rates between the groups of months, patients belonging to group 1 presented a higher overall mortality rate than the other pools (25.1% vs. 20.4%; *p* = 0.014), with a relative risk of 1.23 (95% IC = 1.06 – 1.59). Conversely, patients in group 2 had a lower mortality rate than the others (16.1% vs. 23.3%; *p* < 0.001) with a relative risk of 0.64 (95% IC = 0.49 – 0.82). No differences were found for patients in group 3 (22.4% vs. 21.1%, *p* = 0.47). Stratified analysis of OS according to tumour stages revealed in stage I, group 2 presented higher OS than group 1 (208.44 ± 1.55 vs. 191.35 ± 4.57 months; *p* = 0.003) and, similarly, group 3 had higher OS than group 1 (210.18 ± 1.81 vs. 191.35 ± 4.57 months; *p* = 0.003). While in stage II the group 2 presented better OS than the group 1 (199.36 ± 3.67 vs. 185.17 ± 4.79 months,* p* = 0.019) but similar to group 3 (199.36 ± 3.67 vs. 196.50 ± 3.04 months,* p* = 0.120).

Between-group analysis of recurrence rates showed patients included in pool 1 presented a higher rate than the other pools (22.4% vs. 18.6%; *p* = 0.03). Group assignment relative risk for cluster 1 was 1.21 (95% IC = 1.03 – 1.43). However, no differences in the recurrence rate were found between group 2 and the other pools (16.8% vs. 20.4%; *p* = 0.071), nor between group 3 and the others (19.3% vs. 19.9%; *p* = 0.65). Stratified analysis of DFS according to tumour stages showed in stage I, the group 3 presented better DFS than group 1 (207.04 ± 2.25 vs. 186.49 ± 5.06 months, *p* = 0.002) but similar to group 2 (207.04 ± 2.25 vs. 199.74 ± 3.78 months, *p* = 0.067).

## Discussion

This study presents an innovative approach in identifying month-of-birth groups based on the recurrence and mortality due to cancer progression in CRC patients using non-supervised learning techniques. CRC survival is likely related to patient-dependent factors, tumour features and treatments used. In clinical practice, nonetheless, patients diagnosed with CRC can possess similar prognostic factors, yet develop different oncologic outcomes. This study aimed to provide greater insight into the differing oncologic evolution of CRC patients who presented similar preoperative characteristics.

As an unsupervised classification method to objectively determine the distribution of months of birth we used k-means, a mathematical algorithm which avoided the possibility of researcher bias. Notably, a seasonal pattern was not observed, probably due to the setting of the Mediterranean coastal climate in Spain, where seasons overlap without clear boundaries. Season-of-birth patterns have been identified for many chronic health outcomes and for CRC incidence [11, 14]. The relationship between the month of birth and longevity has also been researched. Population studies based on data for Austria, Denmark, and Australia or for Sweden found that month of birth was a significant predictor of mortality at age above 50 years. The differences in lifespan were independent of seasonal distribution of deaths and social differences in the seasonal distribution of births [2, 15]. In the present study, the k-means statistical classification grouped certain months from different seasons with similar oncologic outcomes. However, the reason for this finding is not clear. The results may raise the question of whether the higher survival of group 2 was the result of the longevity associated with each month or whether these months with higher survival had fewer mortality risk factors. These findings regarding mortality are similar to those obtained in multiple other studies, where individuals born in the last months of the year had higher life expectancy than those born during summer months, which showed the worst longevity [2, 14, 16, 17].

After clustering the months of birth, patient and tumour characteristics showed no statistical differences. The groups could therefore be considered sufficiently similar for comparison to be made between them. Each group had significantly different OS and DFS, as Kaplan–Meier curves proved. Furthermore, this distribution of months was predictive of rate of survival in both cases. Patients born in January, February, June, July, October and December (Group 2) had significantly longer OS and DFS than the other groups, with a 1.56 times higher probability of survival. In contrast, patients born in March, April or May (Group 1) presented the worst outcomes of the series. These findings are in line with those of previous studies on the influence of birth month on the natural evolution of malignant neoplasms [15]. In a United States study including 90,000 patients who died of cancer, the average lifespan of those born in winter was 1.5 years longer than those born in summer, assuming that the observed phenomenon was related to health in general and not cancer in particular [18]. Other studies found no association, although some included heterogeneous patients with different tumours, from different populations and analyses were based on distribution by months or seasons, so conclusions should be drawn with caution [2].

Stratified OS and DFS analysis revealed between-group differences in early stages, but without a clinicopathological pattern that could explain these findings. A larger sample size would probably also show differences in advanced stages. The mechanisms explaining the relationship between month of birth and the natural evolution of CRC remain to be elucidated. It should be noted that our analysis did not prove causality, and that the effect size was very limited. Prospective randomized trials should be necessary to establish a causal relationship between the month-of-birth and the CRC survival, however it impossible to carry out this kind of research. This study suggests that factors related to pregnancy or affecting the early months of life could influence the prognosis of CRC patients.

There is evidence suggesting that risk of cancer and mortality are determined by not only genetics and lifestyle but also factors acting during the prenatal or early postnatal period. Well-established epidemiological and experimental literature support that intrauterine environment plays a major role in health trajectory over the course of a person’s life. Conditions and events in very early pregnancy, corresponding to the time when the colon differentiates from the rectum, may alter the developing foetal tissue in the gastrointestinal tract and contribute to pathology later in life. Nutritional deficiencies during early development may have long-lasting effects on mortality later in life. Recent research suggests that both improper diet and nutrient imbalances may be strong sources of mutagenesis [19, 20]. Exposures to infections in the perinatal period could be another factor involved. It is known that certain strains of *Escherichia coli* responsible for the biosynthesis of the genotoxin colibactin have been linked to CRC oncogenesis. Recent research observed that transmission of the genotoxin appears to occur at a very early life stage in new-borns. Early colonization of the neonate gut with genotoxic *E. coli* could influence intestinal homeostasis at adulthood in a way that puts individuals at risk of CRC and other immune-mediated diseases [21, 22]. Exposure to radiation, nutritional status, ambient temperature or other environmental influences during perinatal months could also promote genetic predisposition to mutations or certain genetic mutations that would contribute the neoplasm formation or determine oncologic prognosis throughout life [23, 24].

The association between chronic inflammatory processes and CRC is well-established and systematic inflammatory response markers are considered the most informative prognostic factors in many types of cancer [25, 26]. Several studies have found that early exposure to environmental factors may induce a chronic inflammatory status and increase the risk of cancer in this population, providing support to the relationship between the month of birth and higher risk of cancer or chronic diseases [7, 27]. Furthermore, it has been convincingly demonstrated that adult immune responses can be programmed by neonatal exposure to certain immune-modulating stimuli throughout the process of priming of the antibody-forming system [28]. Neonatal maturation of the immune system is considered determinant in lifelong health status and essential in cancer survival [29]. The pattern of birth month could involve differing immune maturations during the perinatal period and may explain different CRC outcomes for patients with similar tumour characteristics.

Survival analysis revealed that month of birth could be an independent prognostic factor for CRC. Confirmation of these results in larger population studies would make timing of birth a non-modifiable risk factor in CRC patients, which could have profound implications for clinical practice, public health policies and future research on CRC.

An amazing finding of this study was the identification of month of birth clusters as a potential independent prognostic factor for OS and DFS. The grouping of birth months was obtained through the use of unsupervised classification techniques. To our best knowledge, this is the first study to identify an association between month of birth and CRC progression. The main strength of this study is the statistical analysis used. The mathematical algorithms used in this model permit objective identification of three groups of months with distinct oncological outcomes, regardless of patient oncological characteristics, and avoiding observer bias. The study cohort was homogeneous, with similar management for all patients over a long time period.

This study should be interpreted in the light of certain limitations. It is an observational, single-centre study, conducted in a specific geographical area. While the cohort may be representative of the Spanish population, the results might not be generalizable to other regions of the world.

The relationship between the month of birth and CRC survival is likely to depend on the geographical region and the population under study. Therefore, regions with similar characteristics may potentially confirm our findings. Additionally, the sample size was limited to a health department of about 350,000 inhabitants. A larger sample size could modify the results, so conclusions should be accepted with caution. The calibration and validation of the models revealed more favourable outcomes for OS Cox regression model. However, a small sample size is a limitation for these models, so may be difficult to generalize their findings. Consequently, resampling techniques (bootstrap and cross-validation) were necessary to assess the risk of certain variables underrepresented during sample splitting. Study of socio-economic factors, which could be confounding variables, was beyond the scope of the study. Finally, the mechanisms underlying the impact of birth month on CRC outcomes need further research.

## Conclusion

In conclusion, our results provide evidence of an association between month of birth and OS and DFS outcomes of CRC. Three different risk groups of birth months were identified. Patients born in the months of January, February, June, July, October and December had better OS and DFS than those born in different months of the year.

### Supplementary Information

Below is the link to the electronic supplementary material.

Graph 1. Graphic of internal calibration of OS Cox Regression Model. (PNG 12 kb)

Graph 2. Graphic of internal calibration of DFS Cox Regression Model. (PNG 13 kb)

Supplementary file1 (DOCX 15 kb)

## Data Availability

Data supporting the findings of this study are available from the corresponding author upon reasonable request.
