# Supplementary figures and images for: Does month of birth influence colorectal cancer prognosis?
Source: Langenbecks Arch Surg. 2023 Oct 26;408(1):419. doi: 10.1007/s00423-023-03161-3 (PMC10602963; doi:10.1007/s00423-023-03161-3)

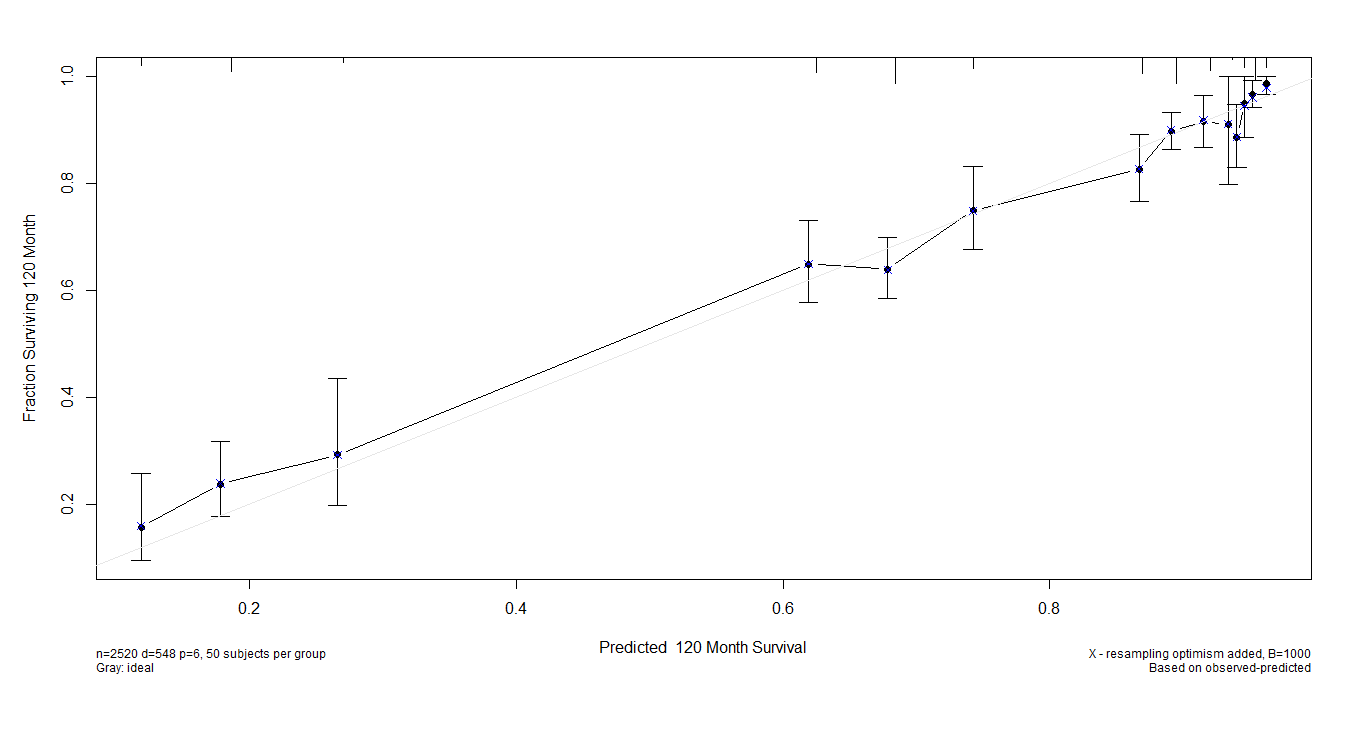

Supplement: Supplementary file 1 — Graph 1. Graphic of internal calibration of OS Cox Regression Model. (PNG 12 kb) [file 423_2023_3161_MOESM1_ESM.png]

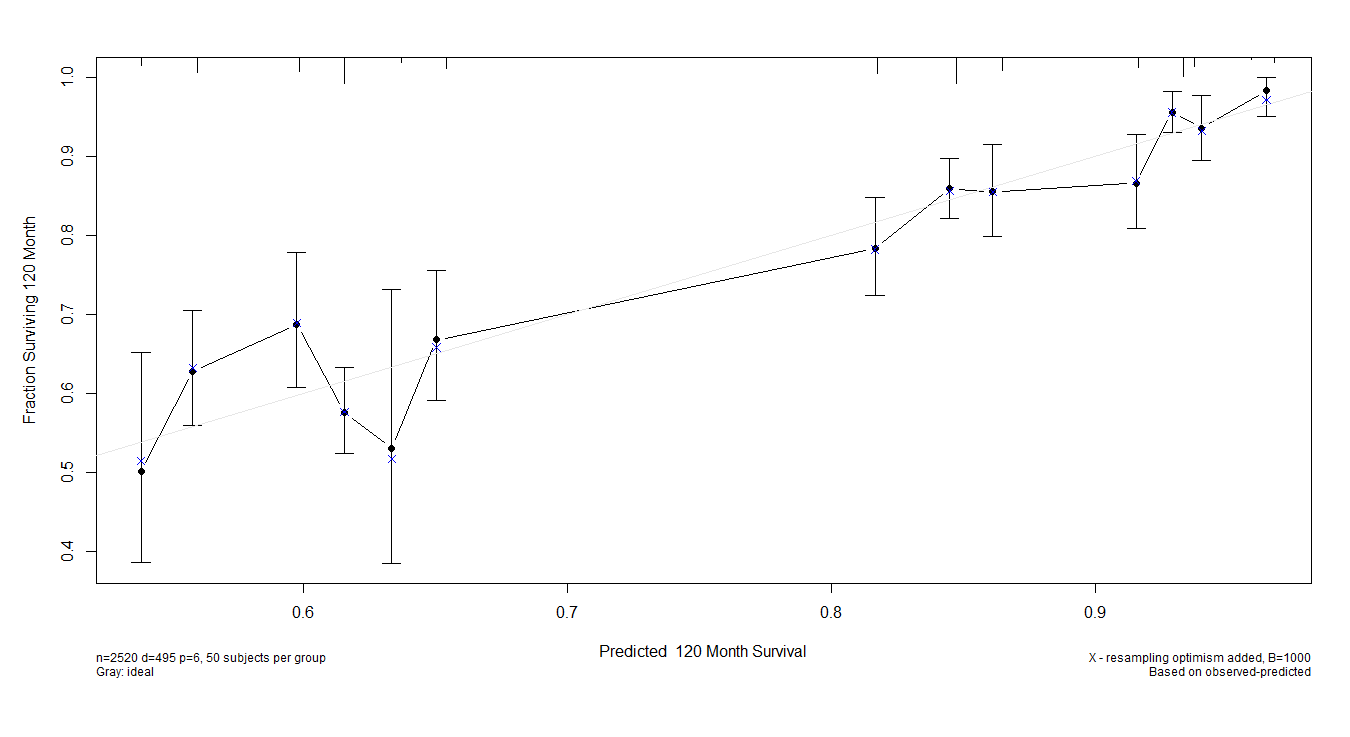

Supplement: Supplementary file 2 — Graph 2. Graphic of internal calibration of DFS Cox Regression Model. (PNG 13 kb) [file 423_2023_3161_MOESM2_ESM.png]
