# Supplementary material for: Does month of birth influence colorectal cancer prognosis?
Source: Langenbecks Arch Surg. 2023 Oct 26;408(1):419. doi: 10.1007/s00423-023-03161-3 (PMC10602963; doi:10.1007/s00423-023-03161-3)
Supplement: Supplementary file 3 — Supplementary file1 (DOCX 15 kb) [file 423_2023_3161_MOESM3_ESM.docx]

SUPPLEMENTARY TABLES

| **Overall Survival Model** | | | | | |
| --- | --- | --- | --- | --- | --- |
|  | Original Index | Training | Test | Optimism | Corrected Index |
| Dxy | 0.6133 | 0.6133 | 0.6199 | -0.0065 | 0.6198 |
| R2 | 0.2625 | 0.2626 | 0.3288 | -0.0662 | 0.3286 |
| Slope | 1 | 1 | 1.0847 | -0.0847 | 1.0847 |
| D | 0.0889 | 0.0892 | 0.1859 | -0.0967 | 0.1856 |
| U | -0.0002 | -0.0002 | 0.0171 | -0.0173 | 0.0171 |
| Q | 0.0892 | 0.0894 | 0.1688 | 0.0794 | 0.1685 |

| **Disease-Free Survival Model** | | | | | |
| --- | --- | --- | --- | --- | --- |
|  | Original Index | Training | Test | Optimism | Corrected Index |
| Dxy | 0.3911 | 0.3912 | 0.4009 | -0.0097 | 0.4009 |
| R2 | 0.1034 | 0.1035 | 0.1468 | -0.0433 | 0.1467 |
| Slope | 1 | 1 | 1.1771 | -0.1771 | 1.1771 |
| D | 0.0347 | 0.0348 | 0.0720 | -0.0373 | 0.0719 |
| U | -0.0003 | -0.0003 | 0.0177 | -0.0180 | 0.0177 |
| Q | 0.0349 | 0.0350 | 0.0543 | -0.0193 | 0.0542 |

**Supplementary Tables. Metrics of Overall Survival and Disease-Free Survival.**

***Dxy*** *(Somers' correlation index),* ***R2*** *(Coefficient of determination),* ***Slope*** *(Indicates the change in the dependent variable for a one-unit change in the independent variable),* ***D*** *(Assesses the model's performance in correctly classifying observations),* ***U*** *(Quantifies how the predicted probabilities match the observed outcomes),* ***Q*** *(Assesses the accuracy of the predicted probabilities and their calibration to the actual outcomes).*
